# Parkinsonism in essential tremor cases: A clinicopathological study

**DOI:** 10.1002/mds.27729

**Published:** 2019-06-10

**Authors:** Ali H. Rajput, Emma F. Rajput, Sarah M. Bocking, Roland N. Auer, Alex Rajput

**Affiliations:** ^1^ Saskatchewan Movement Disorders Program University of Saskatchewan/ Saskatchewan Health Authority Saskatoon Saskatchewan Canada; ^2^ Department of Pathology Saskatchewan Health Authority Saskatoon Saskatchewan Canada

**Keywords:** co‐occurrence, essential tremor, parkinsonism, pathology, resting tremor evolution

## Abstract

**Background:**

Essential tremor and Parkinson's syndrome are two common movement disorders that may co‐occur in some individuals. There is no diagnostic neuropathology for essential tremor, but in PD and other Parkinson's syndrome variants, the neuropathology is well known. The spectrum of Parkinson's syndrome variants associated with essential tremor, their clinical features, and course have not been determined in autopsy‐confirmed cases.

**Objectives:**

To identify: diagnostic features of essential tremor/Parkinson's syndrome, different Parkinson's syndrome variants, and long‐term clinical profile in such cases.

**Methods:**

Patients that had an essential tremor diagnosis and a subsequent clinical or pathological diagnosis of Parkinson's syndrome seen in our clinic during 50 years were included. The diagnosis of parkinsonism was made when bradykinesia, rigidity, and resting tremor were all clinically evident.

**Results:**

Twenty‐one cases were included. All the common variants of parkinsonism co‐occurred with essential tremor. The most common was PD (67%) followed by PSP. The pathological findings were not predicted clinically in 2 cases that had essential tremor/PD and in all 5 essential tremor/PSP cases.

**Conclusion:**

In most essential tremor/Parkinson's syndrome patients, the main motor features of parkinsonism—bradykinesia, rigidity, and resting tremor—were identifiable. All known degenerative Parkinson's syndrome variants co‐occurred in essential tremor patients. © 2019 The Authors. *Movement Disorders* published by Wiley Periodicals, Inc. on behalf of International Parkinson and Movement Disorder Society.

Essential tremor (ET) and Parkinson's disease (PD) are the two most common movement disorders.^1^, ^2^, ^3^, ^4^, ^5^, ^6^ Parkinson's disease (PD) is characterized by marked SN neuronal loss and Lewy body inclusions^7^, ^8^, ^9^, ^10^, ^11^ and is the most common variant of Parkinson's syndrome (PS). The term parkinsonism is used interchangeably with PS. The PSs include PSP, MSA, and Pick's disease.

Both ET and the PD are concentrated in old age.^12^, ^13^ Some elderly individuals may therefore have both ET and PD by chance alone. There is ongoing debate over the frequency of co‐occurrence, clinical diagnosis, course, long‐term clinical profile, different PS variants, and the significance of the co‐prevalence of PS in ET cases.^14^, ^15^, ^16^, ^17^, ^18^, ^19^, ^20^, ^21^, ^22^, ^23^, ^24^, ^25^

Because there are no biological markers for ET or PS, the diagnosis of each is based on the clinical history and findings.^16^, ^26^, ^27^, ^28^, ^29^ There is no diagnostic brain pathology in most ET cases,^16^, ^17^, ^30^ but most PS variants have distinct brain pathology.^9^, ^10^, ^11^, ^31^ Functional imaging, such as PET and dopamine transporter (DaT), studies can distinguish ET from PS cases, but cannot differentiate between PS variants.^32^, ^33^ These imaging studies are valuable for research, but are not widely available and are not essential for general neurology practice.^34^

It is generally assumed that when ET and PD co‐occur, ET manifests first and PD later.^14^, ^18^, ^19^, ^20^ Resting tremor, which is characteristic of PD, may also occur during the late stages in some ET patients.^16^, ^29^, ^30^, ^35^ Action tremor, which is the diagnostic feature of ET, is also present in some PD patients.^36^ Additionally, the clinical features of ET and PD each evolve over time.^16^, ^37^, ^38^, ^39^ These overlapping phenotypes make identification of PD difficult, in ET patients. Bradykinesia and rigidity are two other motor manifestations of PD that are not part of ET. These are therefore important clinical considerations, for a PD diagnosis in ET patients.

Several clinical and epidemiological studies have reported on the ET‐PD co‐prevalence.^6^, ^14^, ^21^, ^23^ Significance of such studies is limited because the brain pathology was not identified. One clinicopathological study suggested an increased risk of PD,^20^ but another reported higher risk of PSP^25^ in ET cases. However, several other clinicopathological studies found no excess risk of PD in ET cases.^15^, ^16^, ^17^ If there were an exaggerated risk of PD or PSP in ET cases, shared genetic etiology would be a major consideration.

Clinical features of ET patients soon after the diagnosis of parkinsonism have been reported.^18^, ^19^ Resting tremor and action tremor severity were greater in the ET‐PD group than in the PD‐only cases.^18^ However, the entire clinical course in autopsied verified cases has not been studied.

We report our observations on longitudinally followed, autopsied ET cases that had a second diagnosis of parkinsonism.

## Materials and Methods

Movement Disorders Clinic Saskatchewan (MDCS) has been conducted uninterrupted by the same one (A.H.R.) or two (A.H.R. and A.R.) movement disorders neurologists since 1968. All residents of Saskatchewan carry a general tax‐funded provincial health care insurance. There is no direct cost to patients to attend the MDCS. Every MDCS patient is referred by a physician. A typical referral letter outlines the patient's problem and the reasons for consultation request. Movement disorders neurologists can follow their patients as they deem appropriate, without new referral request. Longitudinal follow‐up of patients is of MDCS special interest. Patients are generally followed at a 6‐ to 12‐month interval.^40^, ^41^

At each clinic visit, the patient is evaluated by one or both movement disorders neurologists. We ask that one or more family members/caregivers accompany the patient, which adds credibility to the subjective data. There is no time restriction for clinical assessment of a patient at MDCS. More details of this program are provided in an open access publication.^41^

At each clinic visit, severity of motor symptoms of parkinsonism and ET, treatment status, and drug adverse effects are documented. From 1968 to 1987, motor symptoms were evaluated using the Webster scale^42^ and global parkinsonism‐related disability by H & Y scale.^43^ Subsequent to that, the UPDRS motor scale has been used.^44^ To harmonize the 50‐year clinical data, we classified the UPDRS tremor severity score: 1 = mild, 2 = moderate, and 3 and 4 = marked. The Mini‐Mental State Examination is performed, where possible—approximately once a year. Videos are made on all consenting subjects, and sequential videos are made on some patients.

Diagnosis of ET was based on information provided by the referring physician, history provided by the patient/family, and clinical findings at the time of evaluation. ET was diagnosed when the patient had upper limb action tremor, with or without head tremor, which was not attributable to another neurological or systemic disorder.^16^, ^27^, ^29^, ^45^ The second diagnosis of PS and of PD was made when all three major motor features—resting tremor, bradykinesia, and rigidity—were observed.^24^ In some cases, the diagnosis of ET‐PS was made by the referring physician and confirmed by the movement disorders neurologist. In all cases, the clinical features and diagnosis at initial MDCS assessment were noted. If the clinical picture of a patient changed during the follow‐up at MDCS, which required revision to the diagnosis, the new clinical features that resulted in change of diagnosis were noted.

At an opportune time, a patient seen at MDCS is offered the choice of autopsy study at no cost to the family or estate of the patient. The patient is assured that this decision would not impact the ongoing care. It is encouraged that the patient take the declaration form home and discuss it with family before making that decision. This decision may be reversed by the patient at any time. If a patient decides against autopsy, he or she is never asked again. Autopsy is performed within 24 hours of death. The movement disorders neurologists are on 24/7 call for autopsy. The body is transported to the Royal University Hospital in Saskatoon for autopsy. Consent for the autopsy is approved by Saskatchewan Health Authority, and consent for use of brain tissue for research is approved by the Bioethics Board University of Saskatchewan. Immediately after removal, the brain is divided at midline. One half‐brain is fixed in formalin for histological studies and the other half is frozen at –80°C. Neuropathology study is performed by a Canadian certified neuropathologist using stains, including alpha‐synuclein, ubiquitin, and tau immunochemistry, as they became commercially available.^40^ The neuropathologist has full access to patient clinical information at all times. The neuropathologist prepares a detailed report that is shared with the family, and an offer is made to discuss the findings with the neurologist if they so desire.^41^ Final clinicopathological diagnosis is made by the treating neurologist, considering all clinical and neuropathology information. Original patient clinical records, videos, frozen brains, formalin brain remnants, pathology slides, and paraffin blocks are all preserved in our laboratory.^41^

All ET patients seen at MDCS that came to autopsy between 1968 and 2018 were considered. Those patients that had a diagnosis of ET and subsequently developed parkinsonism and those in whom the clinical diagnosis of parkinsonism was not made, but had neuropathological findings of a known variant of parkinsonism, were included in this study. Drug‐induced parkinsonism cases were excluded.^46^

## Results

A total 589 patients followed at MDCS between 1968 and 2018 came to autopsy. Of those, 69 (12%) had a diagnosis of ET. Twenty‐one (30%) of the ET patients that had either an additional clinical, or final clinicopathological diagnosis of ET‐PS, were included in this study. In 5 cases, the diagnosis of ET‐PS made by the referring physician was confirmed at the first MDCS assessment. Table 1 shows individual patient data pertinent to ET and parkinsonism, at the first MDCS visit. Where the initial MDCS diagnosis was revised during follow‐up visits, the reasons are noted in Table 1.

**Table 1:** Clinical and pathology details of each case (n = 21)

Case No. Sex	Site of ET Onset (Age at ET Onset in Years)	First MDCS Visit Since ET Onset in Years (Age at First Visit at MDCS in Years)	Motor Signs at First Visit (Diagnosis)	Family History Tremor, ET, or PD	Age at Second New Diagnosis and Clinical Features	Clinical Diagnosis at Last Visit	Final Clinicopathological Diagnosis (First Visit to Death Interval)
1 Male	Right upper limb (age 53)	15 (age 68)	Lip tremor; action tremor both upper limbs (Mi); rest tremor right upper limb (Mo) (ET + RT)	Father and 1 brother had “tremor”	Age 73 Bradykinesia, rigidity + action tremor, RT	ET + PD	ET + PD (16 years)
2 Female	Head (age 58)	22 (age 80)	Action tremor both upper limbs (Mo); rest tremor (Mi); bradykinesia; rigidity (ET + PD)	Negative	NA	ET + PD	ET No PS pathology, single Lewy body in the amygdala (7 years)
3 Male	Both upper limbs (age 30)	38 (age 68)	Action tremor both upper limbs (Mo); Rest tremor both upper limbs (Mi); dysautonomia; bradykinesia; rigidity (diagnosis confirmed) (ET + MSA)	Father and 1 brother had “tremor”	Age 59 (outside diagnosis of ET + MSA at age 59)	ET + MSA	ET + MSA + PD (7 years)
4 Male	Both upper limbs (age 30)	35 (age 65)	Action tremor both upper limbs (Ma); Rest tremor both upper limbs (Mi) (ET + RT)	Father had “tremor”	Age 73 Cerebellar ataxia	ET + RT + cerebellar ataxia (No Dx of PS)	ET + PD and patchy cerebellar degeneration (15 years)
5 Male	Both upper limbs (age 12)	60 (age 72)	Action tremor both sides (Mo); rest tremor in both upper limbs (Mi); bradykinesia; rigidity (Diagnosis confirmed) (ET + PD)	1 paternal cousin had autopsy confirmed PD^a^	Age 70 (outside diagnosis of PD at age 70—confirmed)	ET + PD	ET + PD (7 years)
6 Male	Right upper limb (age 63)	14 (age 77)	Action tremor both upper limbs (Mo); rest tremor both upper limbs (Mi) (ET + RT)	Negative	Age 78 Gait difficulty; action tremor in the right (Mo) and left (Ma) upper limbs; rest tremor on right (Mo) and left (Ma) upper limbs; bradykinesia; rigidity	ET + PD	ET + PD (5 years)
7 Male	Both upper limbs (age 6)	48 (age 54)	Action tremor both upper limbs (Mo); rest tremor both upper limbs (Mi); rigidity and bradykinesia upper limb (ET + PD)	Negative	Age 53	ET + PD	ET + PD (7 years)
8 Male	Both upper limbs (age 21)	55 (age 76)	Action tremor both upper limbs (Mo); rest tremor both upper limbs (Mi) (ET + RT)	1 brother had “PD”; 1 son tremors; 1 daughter has ET^a^	PS never diagnosed	ET + RT 4 assessments last one was 2 months before death	ET + PD (9 years)
9 Female	Right upper limb (age 55)	25 (age 80)	Action tremor both upper limbs (Mo); rest tremor both upper limbs (Ma); rigidity; bradykinesia (ET + PD)	One aunt had “PD”	NA	ET + PD	ET + PD (9 years)
10 Male	Right upper limb (age 58)	6 (age 64)	Action tremor both upper limbs (Mo); rest tremor upper limb (Mo); head tremor (Mo) (ET + RT)	Negative	Age 68 Action tremor in both upper limbs (Ma); rest tremor both upper limb (Ma); rigidity; bradykinesia	ET + PD Had DBS	ET + PD (7 years)
11 Female	Right upper limb (age 60)	27 (age 87)	Action tremor both upper limbs (Mo); rest tremor upper limb (Ma) and one foot (Mo); bradykinesia; rigidity (Dx confirmed) (ET + PD)	1 sister has “PD”	Age 86 Rest tremor left upper limb (outside diagnosis of PD at age 86)	ET + PD	ET + PD (8 years)
12 Female	Head (age 50)	16 (age 66)	Head tremor (Mo); action tremor both upper limbs (Mo); rigidity; bradykinesia bilateral asymmetrical (ET + PD)	1 brother “PD”; 1 sister “PD”	Age 66	ET + PD	ET + PD (12 years)
13 Male	Both upper limbs (age 15)	56 (age 71)	Action tremor both upper limbs (Mo); rest tremor both upper limbs (Mo) and left leg; bradykinesia; rigidity (ET + PD)	1 brother “tremor”; 1 sister “tremor”; 1 son “tremor”; mother “tremor”	NA	ET + PD	ET + PD (4 years)
14 Male	Right upper limb (age 8)	70 (age 78)	Action tremor both upper limbs (Mi); rest tremor both upper limbs (Mo); bradykinesia; bilateral rigidity (Dx confirmed) (ET + PD)	Negative	Age 70 (outside diagnosis of PD at age 70)	ET + PD	ET + PD (5 years)
15 Female	Head (age 71)	10 (age 81)	Action tremor both upper limbs (Ma); rest tremor (Mi); head tremor (Mi) (ET)	Aunt had “head tremor”	PS never diagnosed	ET + RT	ET + PD (5 years)
16 Female	Both upper limbs (age 50)	8 (age 58)	Action tremor both upper limbs (Mo) (ET)	Mother upper limb “tremor”	Age 64 Dystonia both upper limbs, right more marked than the left; apraxia right upper limb; rest tremor right upper limb (Mo); action tremor both upper limbs (Mo) (Dx: Corticobasal syndrome)	ET + CBS	ET + Pick's disease (10 years)
17 Male	Both upper limbs (age 60)	15 (age 75)	Action tremor both upper limbs (Mo); rest tremor Left upper limb (Mi) (ET)	1 sister ET history but normal brain pathology	Age 87 Decline in writing; rest tremor both upper limb (Mo) and both lower limb (Mi); action tremor (Mo); bradykinesia right more than left; rigidity right more than left	ET + PD	ET + PSP (13 years)
18 Male	Both upper limbs (age 10)	68 (age 78)	Action tremor both upper limbs (Mo); rest tremor both upper limbs (Ma); rest tremor lower limbs (Mi); bradykinesia; rigidity (Dx confirmed) (ET + PD)	1 sister and brother and father and paternal grandfather had “tremor”	Age 72 (outside diagnosis of PD at age 72)	ET + PD	ET + PSP (7 years)
19 Male	Right upper limb (age 66)	5 (age 71)	Action tremor (Ma); Rest tremor both upper limbs (Mi) (ET + RT)	3 brothers and 3 sisters had “tremor”	Age 77 Action tremor both upper limbs (Ma); rest tremor both upper limbs (Mi); rigidity both upper limbs; bradykinesia and rigidity both upper and lower limb	ET + PD	ET + PSP (12 years)
20 Male	Left upper limb and head (age 53)	3 (age 56)	Action tremor both upper limbs (Ma); head tremor (Mo) (ET)	Father had tremor	Age 57 Head tremor (Mo); action tremor upper limb (Mo); bradykinesia; rigidity both sides	ET + PD	ET + PSP (7 years)
21 Female	Age of onset unknown Reported “long duration” of upper limbs action tremor worsening at age of 77	Unknown (age 79)	Action tremor both upper limbs (Mi); rest tremor both upper limbs (Mo); bilateral bradykinesia and rigidity (ET + PD)	Negative	Age 79	ET + PD	ET + PSP (9 years)

Tremor severity: Mi = mild; Mo = moderate; Ma = marked; NA = not applicable; UPDRS +1 = mild; UPDRS +2 = moderate; UPDRS +3 and + 4 = marked; PS = parkinsonism; CBS = cortical basal syndrome; RT = rest tremor.

a Where family member was evaluated at Movement Disorders Clinic Saskatchewan.

Median age of ET onset was 51 (6–71) years. Family history of tremor, ET, or parkinsonism was positive in 15 (71%) cases. This information was historical in most cases (Table 2). In rare cases, the affected family member was assessed at MDCS (Table 1). In most cases, such information was of a general nature, indicating presence of tremor without specific diagnosis. Median follow‐up after first MDCS visit until death/autopsy was 7 (4–16) years. Mean duration of ET until the second diagnosis of PS was 30 (median 24; 4–62) years. Mean survival after ET onset was 38 (10–75) years (Table 2).

**Table 2:** Profile of all ET cases (n = 21)

Sex (n)	M = 14 F = 7
Age at ET onset (years)	Median = 51 Range = (6–71)
Site of ET onset (n)	Only one upper limb = 7; Only head = 3; Both upper limbs = 10; Head and upper limbs = 1
Family history of tremor or ET or PS	15/21 (71%)
Duration of years before second clinical diagnosis (excluding 3 RT)	n = 18 Mean = 30 Range = (4–62) Median = 24
ET + PD clinical diagnosis group—second diagnosis onset features	n = 16 Bradykinesia/gait difficulty 11/16
Final clinical motor profile at final assessment (n)	Mixed = 10 Akinetic/rigid = 5 Tremor predominant = 6
First visit to death duration	Median 7 (4–16) years
Dementia (n)	8/21 Unknown = 2
Clinical diagnosis at last evaluation (n)	ET + PD = 16 ET + CBS = 1 ET + RT = 3 (one with ataxia) ET + MSA = 1
Duration of ET until death (years)	Mean = 38 Range = (10–75)

M, male; F, female; CBS, corticobasal syndrome; RT, rest tremor.

Table 3 is a summary of different clinicopathological diagnostic subgroups. The most common final clinicopathological diagnosis was ET‐PD in 12 cases. Four patients, including 1 with ET‐PD diagnosis, had the earlier tremor‐dominant manifestations change, to akinetic‐rigid during follow‐up.^47^

**Table 3:** Final clinicopathological diagnosis profile (n = 21)

Pathological Diagnosis	PD n = 12	PD + Cerebellar Degeneration n = 1	PSP n = 5	ILB n = 1	Pick's Disease n = 1	MSA + PD n = 1
Clinical diagnosis at last visit	ET + PD (n = 10) ET + RT (n = 2)	ET + RT + ataxia	ET + PD (n = 5)	ET + PD	ET + CBS	ET + MSA
Final clinicopathological diagnosis	ET + PD (n = 12)	ET + PD + cerebellar degeneration	ET + PSP (n = 5)	ET + ILB	ET + Pick's disease	ET + MSA + PD
Family history of ET, tremor or PS	8/12 positive	Positive	4/5 positive	Negative	Positive	Positive
Final motor profile number of cases	AR (n = 2) TD (n = 4) MX (n = 6)	AR	MX (n = 3) TD (n = 2)	MX	AR	AR
Treatment with l‐dopa number of cases	10 treated; 10 improved;	Not treated	2 treated; both benefited	Treated; Improved tremor; no dyskinesia	Treated; no improvement	Treated; no benefit
Survival after ET onset in years	Mean = 44.5; range (16–80); median = 40.5	50	Median = 23; range (9–75) Unknown (n = 1)	30	18	30
Survival after dual clinical diagnosis in years	Median = 6; range (4–13) Never diagnosed with PS clinically (n = 2)	7	Median = 7.5; range (2–13) unknown (n = 1)	8	8	15
Evolution to AR profile	TD → AR = 1 MX → AR = 1	TD → AR	0	0	TD → AR	TD → AR

ILB, incidental Lewy body inclusions; PS, parkinsonism; CBS, corticobasal syndrome; AR, akinetic‐rigid; TD, tremor dominant; MX, mixed.

Video segment 1 shows a 71‐year‐old ET‐PD patient. She had onset of ET at age 50 and the second diagnosis of PD at age 66. Video segment 2 shows an 81‐year‐old ET‐PD case. He had onset of ET at age 21. The PD pathology was not predicted clinically in this case. He was evaluated several times. The last evaluation was 2 months before death, when the clinical picture was similar to that in the video.

The second largest subgroup was ET‐PSP. It included 5 cases. Video segment 3 shows 1 such patient. He had onset of ET at age 60, and a second diagnosis of PD was made at age 87.

The spectrum of motor features at the final MDCS assessment in the ET‐PD cases included: equal severity of resting tremor and bradykinesia/rigidity (mixed; Video, segment 1), tremor dominant (Video, segment 2), or akinetic‐rigid (Video, segments 4a and 4b).

One patient had clinical diagnosis of ET‐PD, but no pathological changes of a known PS variant. The neuropathologist was aware of the clinical diagnosis and conducted an extensive search. Only one alpha‐synuclein–positive Lewy body was found in the amygdala. Other brain areas pertinent to PS were normal (Table 1, case 2).

One patient had ET and prominent cerebellar ataxia as well PD pathology, which was not predicted clinically. Another patient had clinical and pathological diagnosis of MSA. He had additional PD pathology, which was not predicted clinically (Table 1, case 3).

The most common error in predicting the underlying PS pathology was in 5 ET‐PSP cases. Each of those patients was clinically diagnosed as ET‐PD. None of them had ophthalmoplegia. We could not identify other clinical features that distinguished these ET‐PSP cases from ET‐PD cases (Supporting Information Table).

## Discussion

A major objective of neurology practice is making accurate clinical diagnosis; that is, especially applicable to movement disorders where the laboratory diagnostic tools are few and are not widely available. Diagnosis of ET is strictly clinical because there is no specific brain pathology in these patients.^1^, ^6^, ^17^ Pathological findings characteristic of different PSs are, however, well known.^9^, ^10^, ^31^ Thus, the clinical diagnosis of the underlying pathology of PS can be verified with neuropathology studies, for making the definite diagnosis.^9^, ^10^, ^31^, ^48^

Resting tremor is a well‐known feature of PS that also occurs in some ET‐plus cases.^1^, ^16^, ^24^, ^28^, ^29^, ^35^ Action tremor, which is characteristic of ET,^28^, ^29^ is also a feature of PD.^36^ Because of the overlapping tremor manifestations, clinical distinction between ET and PS can be difficult in some cases.^49^, ^50^ Clinical diagnosis of PD in ET cases is even more challenging. Bradykinesia and rigidity are two major features of PS that are not part of the ET spectrum. Therefore, we used resting tremor, bradykinesia, and rigidity to make the second diagnosis of PS in ET patients.

Seventeen of 21 (81%) of our patients with known parkinsonian pathology were clinically recognized as having PS (Table 3). That figure is higher than reported in one study, where the patients were evaluated at multiple centers.^25^ Only 3 of the 11 (27%) cases that had PSP pathology were recognized as having PS. A closer look at their data^25^ shows that presence of bradykinesia and rigidity were the clinical features that helped make the additional diagnosis of PS.

Motor functions slow with normal aging. That may be mistaken as bradykinesia. In pre‐existing ET and especially those who also have resting tremor, that may be mistaken for PD. The age‐related slowing is symmetrical.^51^ Asymmetrical bradykinesia, rigidity, and resting tremor is therefore a valuable indicator of PD in the elderly ET patients.^31^, ^51^

Response to treatment and the prognosis vary in different PSs.^52^ In general, there is greater benefit on levodopa and the prognosis is more favorable in PD than in PSP and MSA—the two other common Parkinson's variants.^52^ Therefore, every effort is made to identify PD patients clinically.

Accuracy of clinical diagnosis of early‐stage PD, reported in de novo patients, varies between 38% and 65%.^11^ Sixteen of our 21 ET patients had a second clinical diagnosis of PD, which was confirmed at autopsy in 10 (63%; Table 3). The underlying pathology of all PS variants was accurately predicted in 12 of 21 (57%) patients. The most common error was a clinical diagnosis of PD in 5 patients who had PSP pathology. Similar errors have been reported by several other studies.^11^, ^25^, ^53^ The main reason for that is the absence of ophthalmoplegia and early gait difficulty.^53^ Recent literature indicates that PSP is more common than has been recognized so far.^11^, ^53^ Such errors would likely continue until there are biomarkers that distinguish PD from PSP.

How the co‐occurrence of PD in ET cases changes the clinical picture has been reported in some studies.^18^, ^19^ Ryu and colleagues^18^ reported that, with the onset of parkinsonism, these cases developed more pronounced action tremor and resting tremor. Minen and Louis^19^ reported that all ET patients who developed parkinsonism had resting tremor and 94% to 96% had bradykinesia and rigidity respectively. Neither of these studies reported the entire clinical course as we have done. Our patients had a median follow‐up that was 7 (4–16) years (Table 2) from the first MDCS visit until death. In some of our patients, who did not have evidence of PS at first assessment, but developed the parkinsonism during follow‐up, we observed that both the resting tremor and the action tremor were more pronounced at the time of second diagnosis (Table 1), confirming the observations of a previous study.^18^ During follow‐up visits, the clinical picture evolved in some cases. The resting tremor remained unchanged, declined, or fully resolved as seen in Video segments 4a and 4b. We did not observe a consistent pattern of change in action tremor in ET‐PD cases during MDCS follow‐up.

It has been reported that in the ET cases, PD more often starts on the side that has the most pronounced tremor.^6^ We could not validate that. The first symptom of ET was reported as more common in the right than the left upper limb in our cases. One possible explanation is that most individuals are right handed and preferentially use that hand for daily life activities. They are more likely to note right than left upper limb action tremor.

There are conflicting data about which PS variant is more common in ET cases. One clinical study observed that PD cases contrasted to PSP patients were more likely to have a previous diagnosis of ET.^54^ Another report by the same group concluded that the risk of PSP was higher than of PD, in the ET cases.^25^ The most common form of PS in our patients was PD, followed by PSP. Such relative frequency of PS variants is similar to that reported in other large autopsy series of PS.^11^, ^53^ Our entire autopsy cohort has a similar pattern.^41^ Our data show that all the common PS variants co‐occur with ET. Which ET cases are predisposed to one or other Parkinson's variants needs further studies.

Family history of tremor, ET, or parkinsonism was positive in the majority (71%) of our cases. The significance of that cannot be interpreted accurately, given that the data, in most cases, are based on the information provided by patients/families and did not include specific diagnosis. Only a small number of family members with those disorders were evaluated by us (Table 1).

Our study has some limitations. It is not a prospectively designed study to answer specific questions. It includes a small number—21 cases. The findings in our cases may not be applicable to all ET patients, because of referral and autopsy consent bias. The upper limb action tremor, which is typical of ET, can also be an early feature of PD, PSP, and other PS variants. Action tremor in such cases, without other motor feature of parkinsonism, cannot be distinguished from ET. We were unable to identify the optimal duration of isolated action tremor, before the second diagnosis of PS is entertained. We could not make a clinical diagnosis of PD in 2 cases that had prominent resting tremor but no bradykinesia or rigidity (Video, segment 2). In light of that, we recommend that, where possible, such ET patients have a PET or DaT study to distinguish PS form ET. Where that option is not available, the patient should be given a trial on l‐dopa. We cannot explain the reason for some ET‐PD patients remaining tremor dominant and others becoming akinetic‐rigid. We are unable to identify the clinical features of ET cases that are predisposed to PD. The family history in our cases is not precise. We did not perform genetic studies.

However, there are several strengths. This study is based on real‐life practice of neurology. We have identified all the common degenerative Parkinson's variants in ET cases. Our patients had long follow‐up and documented evolution of clinical features with time, and we have reported on the outcomes. It is the largest clinicopathological study of ET‐PS cases to date.

In summary, in most ET cases, the PS manifests as bradykinesia, rigidity, and resting tremor. Except for PSP, most other PS variants can be clinically distinguished from PD. Clinical features of ET‐PD cases evolve with time; resting tremor may resolve completely. Many PSP cases do not have ophthalmoplegia. The most common Parkinson's variant that co‐occurs with ET is PD. The relative frequency of different PSs in this study is similar to that in unselected autopsy series.

The subspecialty clinics like ours offer an opportunity to conduct in‐depth studies, but the patients enrolled are highly selective. Biomarkers of PD, ET, and PSP are needed to study the shared risk for ET and PS.

## Author Roles

(1) Research Project: A. Conception and Design; B. Acquisition of Data; C. Analysis and Interpretation of Data; (2) Manuscript: A. Writing of the First Draft, B. Review and Critique; (3) Other: A. Patient Assessment, B. Data Abstraction, C. Video Preparation, D. Data Storage, E. Data Retrieval, F. Literature Search, G. Neuropathology Studies, H. Patient Assessment, I. Director of Saskatchewan Movement Disorders Program.

A.H.R.: 1B, 2A, 3A, 3B, 3H

E.F.R.: 1C, 2A, 3B, 3C

S.B.: 2B, 3D, 3E, 3F

R.A.: 2B, 3G

A.R.: 1B, 2B, 3H, 3I

## Financial Disclosures

Dr. Alex Rajput has received research support from the Dr. Ali Rajput Endowment for Parkinson's Disease and Movement Disorders.

## Supporting information


**Supplementary table** Comparison of ET‐PD and ET‐PSP Cases (*n* = 17)

**Video** Segment 1: female at age 71. She had ET onset at age 50 and a second diagnosis of parkinsonism was made 16 years later. This video shows head tremor, prominent postural, and resting limb tremor. The left armswing is markedly reduced indicating bradykinesia, and there was more rigidity on that side. Pathology indicated Lewy body PD. Segment 2: male who had essential tremor onset at age 21. This video was taken at age 81. It shows prominent tremor, but there is no bradykinesia. There was no rigidity. This patient had a clinical diagnosis of ET plus resting tremor; autopsy revealed Lewy body PD. Segment 3: patient who had essential tremor onset at age 60 and second clinical diagnosis of PD at age 87. Video was taken at age 87. He has prominent head tremor, postural tremor and kinetic tremor, and bradykinesia. He had rigidity in all limbs. He had pathology evidence of PSP. Segment 4a. Patient had onset of ET at age 53 and second diagnosis of PD made at age 73. This video was taken at age 74. It shows prominent resting tremor, and there was bilateral bradykinesia and rigidity on both sides. Segment 4b is the same individual 9 years later. He has no resting tremor. He had bilateral bradykinesia and rigidity. This case illustrates evolution from tremor‐dominant to akinetic/rigid motor profile.
