# Supplementary material for: Parkinsonism in essential tremor cases: A clinicopathological study
Source: Mov Disord. 2019 Jun 10;34(7):1031–40. doi: 10.1002/mds.27729 (PMC6771898; doi:10.1002/mds.27729)
Supplement: Supplementary file 1 — Supplementary table Comparison of ET‐PD and ET‐PSP Cases (n = 17) [file MDS-34-1031-s001.docx]

**Supplementary table: Comparison of ET-PD and ET-PSP Cases (*n* = 17)**

|  | ET + PD (*n* = 12)  (2 not diagnosed clinically) | ET + PSP (*n* = 5) |
| --- | --- | --- |
| Sex | M = 8 | M = 4 |
| Age of ET onset (years) | Median = 52  Range = (6 - 71) | Median = 57  Range = (10 - 66) |
| Family history of ET / tremor / PD | 8 /12  (67%) | 4/5  (80%) |
| Accurate clinical prediction of pathology diagnosis | 10/12  (83%) | 0/5  (0%) |
| Survival after 2^nd^ diagnosis (years) | Median = 9  Range = (3 – 21) | Median = 6  Range = (1 – 13) |
| First PS sign (historical) | Bradykinesia/gait difficulty = 5/10  Lower limb resting tremor = 2/10 | Bradykinesia = 4/5  Lower limb resting tremor = 1/5 |
| Response to Levodopa | 10/10 improved | 2/2 improved |
| ET duration until 2^nd^ diagnosis of PS (years) | Median = 23  Range = (10 - 62) | Median = 19  Range = (4 – 62) |

**ET = Essential Tremor; PD = Parkinson’s disease; PSP = progressive supranuclear palsy; M = male; PS = Parkinson syndrome**
